# Phenotypic selection of a wild *Saccharomyces cerevisiae* strain for simultaneous saccharification and co-fermentation of AFEX™ pretreated corn stover

**DOI:** 10.1186/1754-6834-6-108

**Published:** 2013-07-27

**Authors:** Mingjie Jin, Cory Sarks, Christa Gunawan, Benjamin D Bice, Shane P Simonett, Ragothaman Avanasi Narasimhan, Laura B Willis, Bruce E Dale, Venkatesh Balan, Trey K Sato

**Affiliations:** 1 Biomass Conversion Research Laboratory (BCRL), Department of Chemical Engineering and Materials Science, Michigan State University, 3900 Collins Road, Lansing, MI 48910, USA; 2 DOE Great Lakes Bioenergy Research Center, Michigan State University, East Lansing, MI 48824, USA; 3 DOE Great Lakes Bioenergy Research Center, University of Wisconsin-Madison, 1552 University Ave, Madison, WI 53726, USA; 4 Department of Bacteriology, University of Wisconsin-Madison, 1550 Linden Dr, Madison, WI 53706, USA; 5 U.S. Department of Agriculture, Forest Products Laboratory, 1 Gifford Pinchot Dr, Madison, WI 53726, USA

**Keywords:** Thermo-tolerance, Xylose fermentation, *S. cerevisiae*, SSCF, AFEX, Degradation products, Ethanol

## Abstract

**Background:**

Simultaneous saccharification and co-fermentation (SSCF) process involves enzymatic hydrolysis of pretreated lignocellulosic biomass and fermentation of glucose and xylose in one bioreactor. The optimal temperatures for enzymatic hydrolysis are higher than the standard fermentation temperature of ethanologenic *Saccharomyces cerevisiae*. Moreover, degradation products resulting from biomass pretreatment impair fermentation of sugars, especially xylose, and can synergize with high temperature stress. One approach to resolve both concerns is to utilize a strain background with innate tolerance to both elevated temperatures and degradation products.

**Results:**

In this study, we screened a panel of 108 wild and domesticated *Saccharomyces cerevisiae* strains isolated from a wide range of environmental niches. One wild strain was selected based on its growth tolerance to simultaneous elevated temperature and AFEX™ (Ammonia Fiber Expansion) degradation products. After engineering the strain with two copies of the *Scheffersomyces stipitis* xylose reductase, xylitol dehydrogenase and xylulokinase genes, we compared the ability of this engineered strain to the benchmark 424A(LNH-ST) strain in ethanol production and xylose fermentation in standard lab medium and AFEX pretreated corn stover (ACS) hydrolysates, as well as in SSCF of ACS at different temperatures. In SSCF of 9% (w/w) glucan loading ACS at 35°C, the engineered strain showed higher cell viabilities and produced a similar amount of ethanol (51.3 g/L) compared to the benchmark 424A(LNH-ST) strain.

**Conclusion:**

These results validate our approach in the selection of wild *Saccharomyces cerevisiae* strains with thermo-tolerance and degradation products tolerance properties for lignocellulosic biofuel production. The wild and domesticated yeast strains phenotyped in this work are publically available for others to use as genetic backgrounds for fermentation of their pretreated biomass at elevated temperatures.

## Background

Recently, fuel ethanol production from lignocellulosic biomass has gained significant interest due to both environmental and social sustainability benefits [1-3]. Cellulosic ethanol has been envisaged to be produced by fermentation of simple sugars from enzymatically hydrolyzed plant biomass. Since the efficiency and rates of enzymatic hydrolysis and fermentation are often optimal at distinct temperatures, separate hydrolysis and fermentation (SHF) is commonly employed to carry out the two reactions in separate vessels, which increase capital costs and total processing time. Simultaneous saccharification and co-fermentation (SSCF) is an alternative process that encompasses two reactions within the same vessel. SSCF is more favorable for cellulosic ethanol production than SHF due to its lower cost, shorter processing time, higher sugar conversions, higher ethanol yields and lower contamination risk [4,5]. An ideal SSCF process would occur at temperatures for optimal cellulolytic activities (e.g., 50°C for commonly-used fungal *Trichoderma reesei* cellulases), which is significantly above the standard culturing temperature of 30°C for *Saccharomyces cerevisiae*, the most commonly used organism for the production of fuel ethanol. As a result, SSCF has been conducted at lower temperatures, slowing enzymatic hydrolysis and sugar release rates and resulting in reduced fermentation rates and yields [5,6].

A critical process step in the conversion of lignocellulosic feedstocks into biofuel is biomass pretreatment. Although biomass pretreatment dramatically increases enzymatic hydrolysis rates and yields, it also results in the formation of degradation products that impair fermentation [7-10]. For instance, the dilute acid pretreatment generates degradation products such as 5-hydroxymethyl-2-furaldehyde (HMF), furfural, acetic acid, and phenolics, all of which affect microorganism fermentation and reduce ethanol yield and productivity [11]. Ammonia fiber expansion (AFEX™) pretreatment generates less inhibitory compounds compared to dilute acid pretreatment due to its mild pretreatment conditions and ammonolysis reactions [12]. However, the major degradation products of AFEX, including acetamide, feruloyl amide, coumaroyl amide [13], still inhibit fermentation by *S. cerevisiae*[8].

Lignocellulosic biomass typically contains 15-35% of hemicellulose, which is primarily composed of xylose [14]. Efficient conversion of such xylose, together with cellulose-derived glucose, into ethanol is also crucial for producing high fuel yields that provide greater return on investment. During the past decades, *S. cerevisiae* has been extensively engineered to ferment xylose [10,15,16]. Two xylose metabolism pathways, xylose reductase (XR)-xylitol dehydrogenase (XDH) pathway [10] or xylose isomerase pathway [15], have been constructed in *S. cerevisiae* resulting in promising xylose fermentation properties. The expression of the XR-XDH pathway genes from the xylose-fermenting yeast *Scheffersomyces stipitis* (historically called *Pichia stipitis*) into industrial *S. cerevisiae* strains has conferred effective xylose fermentation from defined lab media [15,17,18]. In contrast to lab media, the presence of inhibitors generated from biomass pretreatment have significant impact on the fermentation of hydrolysate sugars, particularly xylose. For example, the *S. cerevisiae* 424A(LNH-ST) strain (424A), which was genetically modified to express multiple copies of *S. stipitis XR* and *XDH* genes, as well as endogenous xylulokinase (*XK*) [10] can rapidly ferment xylose in standard yeast extract and peptone (YEP) lab medium, but displayed significantly reduced growth and xylose consumption during SHF and SSCF of AFEX treated biomass [5,6,8].

An efficient SSCF process that converts pretreated lignocellulosic biomass into ethanol requires a microbial strain that could tolerate both high temperature and degradation products and meanwhile maintain efficient xylose fermentation. At present, others have identified *S. cerevisiae* strains capable of effectively fermenting glucose from pretreated lignocellulosic hydrolysates at elevated temperatures [19-21]. However, no work has been published reporting the specific creation of a *S. cerevisiae* strain that can ferment xylose from pretreated biomass and at elevated temperatures, two requirements for effective SSCF processes. Since environmental stresses can impact the rate and yield of xylose fermentation, utilizing a stress-tolerant ethanologenic strain background may make a significant difference in the feasibility and profitability of cellulosic biofuel process. Previous approaches have focused on engineering industrial *S. cerevisiae* strains with robust properties for xylose metabolism [10,11,15,22,23]. However, this approach relies on general stress tolerance properties that may or may not be optimal for the specific media and fermentation conditions of interest. Because optimal SSCF of AFEX pretreated corn stover (ACS) simultaneously imposes both thermal and inhibitory stresses upon the ethanologen, we sought to perform a more comprehensive evaluation of 108 wild and domesticated *S. cerevisiae* strains by phenotyping for growth tolerance specific to simultaneous elevated temperature and inhibitory compounds in ACS hydrolysate (ACSH). These strains were collected from a variety of ecological niches and display a range of phenotypic traits due to their genetic diversity [24-26]. We postulated that strains growing relatively well in ACSH at elevated temperatures would, after directed engineering of the XR-XDH-XK genes, also perform relatively well in SSCF of ACS. Moreover, because these strains are publically available, other researchers can use the phenotypic data to independently develop yeast strains for SSCF of ACS or related pretreated biomass containing similar inhibitors.

## Results

### Phenotyping of wild *S. cerevisiae* strains for AFEX and thermo-tolerance

**Figure 1:**
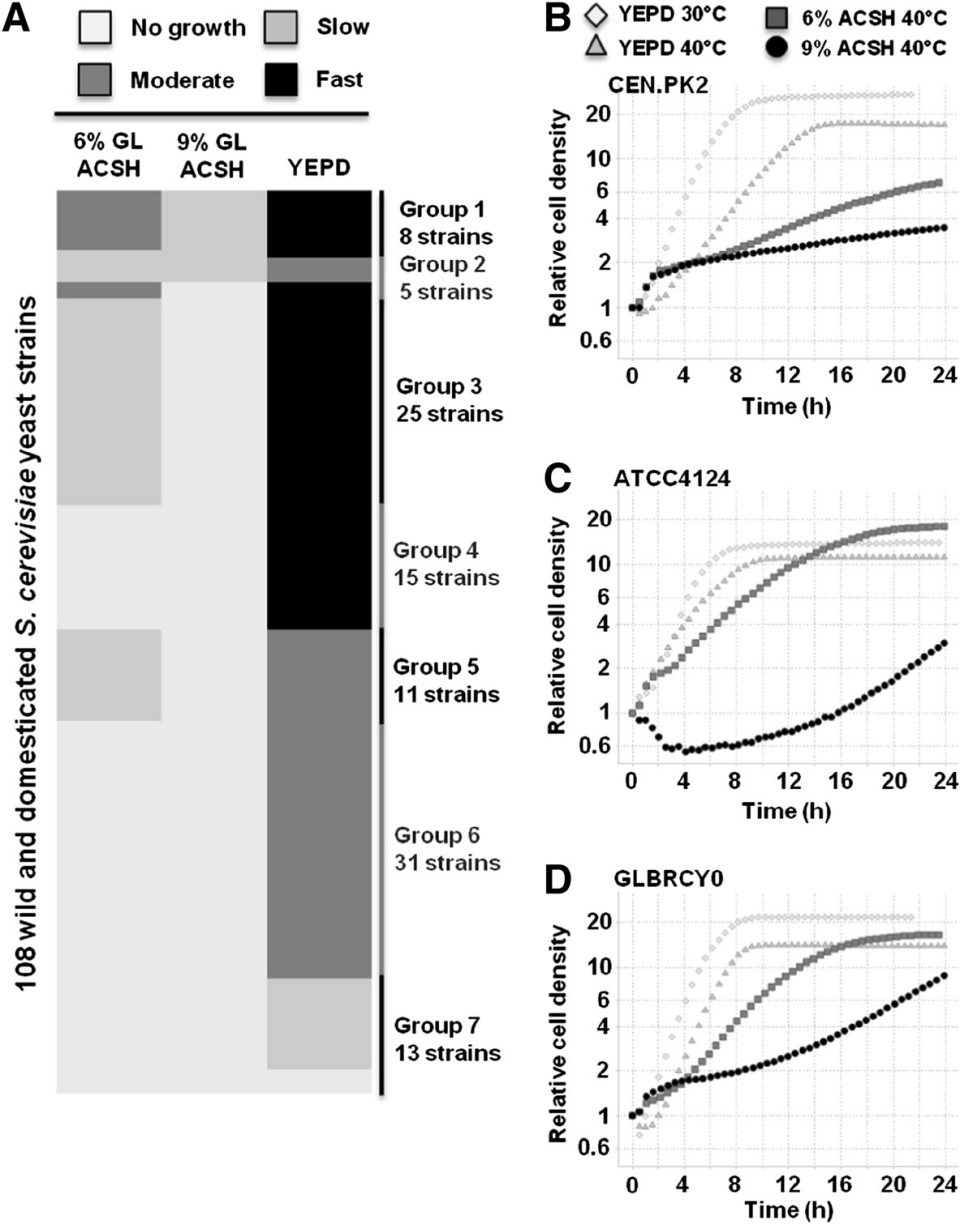
**Identification of *S. cerevisiae *strains with thermotolerance in AFEX corn stover hydrolysate (ACSH). (A)** Heat map displaying binned growth phenotypes of 108 wild and domesticated *S. cerevisiae* strains grown in the indicated media at 40°C. Yeast strains were grown in 96-well plate format on 6% or 9% glucan loading (GL) ACSH at 40°C, or YEPD at 30 or 40°C for 24 hours. Cell growth was continuously monitored by measuring the optical density at 595 nm every 10 minutes with Tecan 96-well plate readers. Specific growth rates in ACSH or YEPD at 40°C were normalized relative to YEPD at 30°C, and resulting values were binned (0–0.2 = “No growth,” 0.2-0.4 = “Slow” growth, 0.4-0.8 = “Moderate” growth, >0.8 = “Fast” growth). Strains were placed into numbered cluster groups (1–7) and the number of strains within each group are indicated along the right side of the heat map based on phenotype and is indicated in the Additional file 1: Table S1. Representative 96-well growth curve data of CEN.PK2 **(B)**, ATCC4124 **(C)** or GLBRCY0 **(D)** in indicated media conditions. Relative cell densities are expressed as background subtracted OD595 values divided by the initial OD_595_ value for each culture. Time points every 30 minutes are shown for clarity.

To identify *S. cerevisiae* strains that can tolerate ACS degradation products at elevated temperatures, we monitored the cell densities of 108 unique wild, domesticated or industrial isolates and laboratory control strains (Additional file 1: Table S1) cultured in 96-well plates containing YEPD medium at both 30 and 40°C or 6% and 9% glucan loading (corresponding to 15.8% and 23.7% solids loading, respectively) ACSH at 40°C. Cell densities from individual wells were used to determine the average specific growth rate from three biological replicates. Average specific growth rates were binned into qualitative assessments of growth rate (no or minimal, slow, moderate, or fast growth rate; Figure 1A). While all or most strains doubled four to five times in cell density at 30 and 40°C in YEPD medium within 24 h, the majority of strains grew much slower in 6% and 9% glucan loading ACSH and did not reach saturation within 24 h, while doubling their cell densities one to two times. The commonly used lab strain, CEN.PK2, is one such strain that grew well in YEPD media, but not in ACSH at 40°C (Figure 1B). Almost half of the strains, including ATCC4124 (Figure 1C), which is the original ancestor of the xylose-fermenting 424A benchmark strain, displayed moderate to fast growth rates in 6% glucan loading ACSH at 40°C but minimal growth in 9% ACSH. In contrast, CBS7960, DBVPG6040, GLBRCY0, IL-01, NC-02, PE-2, PW5, T7, UWOPS83-787.3, UWOPS87-2421 and YJM451 strains all displayed slow but detectable, moderate and fast growth rates in 9% ACSH, 6% ACSH and YEPD, respectively, at 40°C (Additional file 1: Table S1). One wild strain isolated from a banana, which we designated GLBRCY0, displayed robust growth in YEPD and 6% glucan loading ACSH, and slow but significant growth in 9% ACSH at 40°C (Figure 1D). We previously engineered this strain for xylose metabolism in another study [27], thus we opted to further investigate this strain for use in SHF and SSCF with ACS at elevated temperatures.

### Genetic engineering of the wild GLBRCY0 strain for xylose metabolism

The thermo-tolerance of the GLBRCY0 strain in ACSH suggested that it may be able to perform SSCF of ACS at temperatures closer to optimal for saccharification by *Trichoderma reesei* enzymes (50°C). Previously, we stably engineered the GLBRCY0 strain with a single copy of a DNA cassette that conferred heterologous expression of the *S. stipitis* XR-XDH-XK pathway genes, *XYL1*, *XYL2* and *XYL3*, and the modified strain named GLBRCY2A (Y2A), could metabolize xylose from lab media [27]. Previous studies have indicated that over-expression of the *XYL1, 2 and 3* genes can improve xylose metabolism by increasing the flux of xylose into the Pentose Phosphate Pathway [28-30]. Therefore, we generated a diploid GLBRCY0 strain with two confirmed copies of the *XYL1/2/3* expression cassette (*SstiXYL123*) by sporulation and mating, resulting in the diploid GLBRCY35 (Y35) strain.

To examine whether two copies of the *SstiXYL123* gene cassette improved xylose metabolism over one copy in a diploid strain, growth of the engineered GLBRCY0 strains in YEPX media was assayed at 30 and 40°C in microtiter plates. The diploid Y35 strain with two copies of the *SstiXYL123* genes displayed a significant improvement in the rate of growth on xylose at 30°C compared to the diploid Y2A strain engineered with a single copy of the *SstiXYL123* cassette (Additional file 2: Figure S1A). Importantly, both strains carrying at least one copy of the *SstiXYL123* cassette grew significantly faster on xylose than the strain (GLBRCY1A or Y1A) engineered with an empty DNA cassette not containing the *SstiXYL123* genes. At 40°C, all *SstiXYL123* engineered strains maintained significantly faster growth on xylose relative to the control Y1A strain, however there was no difference between strains containing one or two copies of the *SstiXYL123* genes (Additional file 2: Figure S1B). To determine if the higher *SstiXYL123* copy number resulted in corresponding increases in enzymatic activities, we compared the *in vitro* specific *XYL1*/ XR and *XYL2*/XDH activities between strains. With the XR activity assay, almost three-fold higher specific activity was observed from Y35 cell extracts with NADPH relative to Y2A cell extracts, while little activity was observed from Y1A extracts (Additional file 2: Figure S1C). As reported elsewhere for the preferred cofactor of *SstiXYL1*[31], greater activity was observed with NADPH than NADH. With the XDH activity assay, 60% higher specific activity was detected from Y35 cell extracts with NAD^+^, the preferred cofactor for *SstiXYL2*[32], compared to Y2A cell extracts (Additional file 2: Figure S1D). Importantly, little XDH activities were detected in Y1A extracts, or from Y2A and Y35 extracts with NADP^+^. These results indicate that genomic integration of two copies of the *SstiXYL123* correlates with faster growth on xylose at 30°C, due in part to higher *SstiXYL1* and *XYL2* activities, as compared to expression of a single copy of *SstiXYL123* in the same diploid GLBRCY0 background.

### Effect of temperature on fermentation performance in lab media

**Figure 2:**
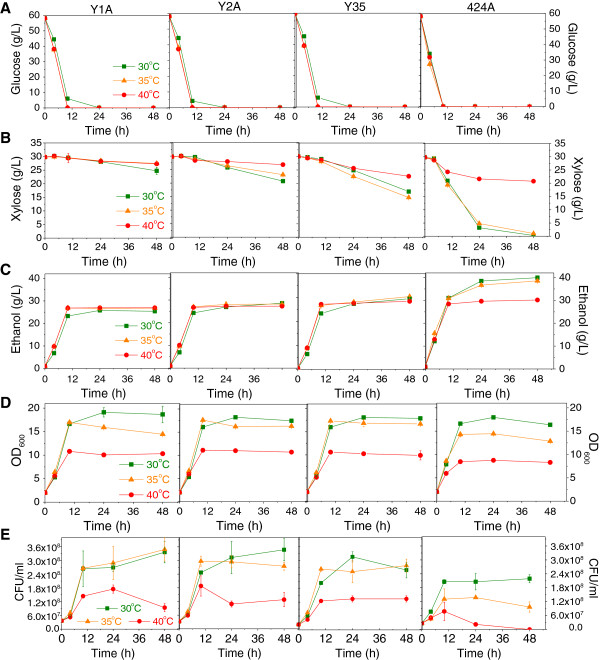
**Fermentation performance comparison of Y1A, Y2A, Y35 and 424A on YEP medium at 30°C, 35°C and 40°C.** Graphs display glucose **(A)**, xylose **(B)**, ethanol **(C)**, cell biomass **(D)**, and viable cell density **(E)** profiles of the four strains during fermentation. Fermentation results with 424A at 30°C are adapted from [7].

While the growth analysis indicated that the engineered GLBRCY0 strains (Y2A and Y35) can metabolize xylose aerobically from YEP media at 30 and 40°C, it remained unclear how well the strains could ferment glucose and xylose at different temperatures. To determine this, we performed fermentation experiments with the Y1A, Y2A and Y35 strains, alongside the 424A strain, at 30 [7], 35 and 40°C in YEP medium containing 58.2 g/L glucose and 29.8 g/L xylose (Figure 2 and Table 1). Cell growth mostly occurred during glucose fermentation while conversion of xylose to ethanol primarily occurred during the stationary phase. Cell growth profiles for all of the strains at 30°C were very similar (Figure 2D). At 35°C, both overall cell growth (Figure 2D) and viable cell densities (Figure 2E) of 424A strain were significantly lower compared to 30°C, however the temperature increase from 30°C to 35°C had no significant impact on Y2A and Y35 strains. At 40°C, Y1A, Y2A and Y35 reached higher maximum cell densities compared to 424A (Figure 2D). Additionally, viable cell densities of Y1A, Y2A and Y35 at all tested temperatures were higher compared to 424A (Figure 2E). Moreover, significantly higher cell viabilities (*p* < 0.05) were observed for Y1A, Y2A and Y35 strains compared to the 424A strain after 48 h. The cell viability of 424A decreased rapidly after exponential growth phase at 40 °C. These results are consistent with our observation that the GLBRCY0 strain background is more thermo-tolerant than the ATCC4124 strain, which is the parental strain of 424A.

**Table 1:** **Fermentation results summary of *S. cerevisiae *strains Y1A, Y2A, Y35 and 424A in YEP medium**^**a**^

	**Y1A (30°C)**	**Y1A (35°C)**	**Y1A (40°C)**	**Y2A (30°C)**	**Y2A (35°C)**	**Y2A (40°C)**	**Y35 (30°C)**	**Y35 (35°C)**	**Y35 (40°C)**	**424A (30°C)**	**424A (35°C)**	**424A (40°C)**
Xylose consumption	17.0% ± 4.5%	7.8% ± 1.3%	8.3% ± 0.5%	30.4% ± 2.4%	22.4% ± 1.4%	9.9% ± 0.3%	43.5% ± 0.6%	50.9% ± 1.4%	24.5% ± 0.4%	99.0% ± 0.0%	96.5% ± 0.0%	30.3% ± 1.1%
Maximum cell biomass (g/L)	9.2 ± 0.5	8.1 ± 0.0	5.2 ± 0.1	8.7 ± 0.1	8.4 ± 0.1	5.3 ± 0.1	8.7 ± 0.0	8.3 ± 0.0	5.1 ± 0.2	8.6 ± 0.0	6.9 ± 0.0	4.2 ± 0.0
Yx/s^b^	0.16 ± 0.01	0.14 ± 0.00	0.09 ± 0.00	0.15 ± 0.01	0.15 ± 0.00	0.09 ± 0.00	0.15 ± 0.00	0.14 ± 0.00	0.09 ± 0.00	0.15 ± 0.00	0.12 ± 0.00	0.07 ± 0.00
Xylitol (g/L)	3.9 ± 0.8	2.1 ± 0.2	2.4 ± 0.0	2.7 ± 0.6	3.2 ± 0.2	1.8 ± 0.0	4.1 ± 0.1	4.2 ± 0.2	2.8 ± 0.2	1.7 ± 0.2	2.6 ± 0.0	0.9 ± 0.0
Glycerol (g/L)	2.7 ± 0.3	2.8 ± 0.0	2.9 ± 0.0	3.5 ± 0.0	3.2 ± 0.0	3.2 ± 0.0	3.8 ± 0.0	3.8 ± 0.0	3.6 ± 0.0	4.7 ± 0.1	6.2 ± 0.1	5.3 ± 0.1
Specific xylose consumption rate (g/h/g cell), 18 h^c^	0.012 ± 0.005	0.011 ± 0.007	0.016 ± 0.026	0.033 ± 0.001	0.022 ± 0.005	0.006 ± 0.003	0.036 ± 0.002	0.048 ± 0.002	0.044 ± 0.012	0.153 ± 0.000	0.151 ± 0.005	0.046 ± 0.002
Ethanol metabolic yield (%)	75.6% ± 3.4%	83.6% ± 1.1%	83.8% ± 0.9%	80.5% ± 0.1%	82.3% ± 0.6%	84.2% ± 0.4%	79.6% ± 0.1%	79.6% ± 0.2%	83.2% ± 0.0%	87.0% ± 0.8%	84.6% ± 0.0%	85.1% ± 0.3%
Ethanol (g/L)	25.4 ± 0.6	26.8 ± 0.2	26.9 ± 0.2	28.6 ± 0.3	28.2 ± 0.0	27.3 ± 0.1	29.6 ± 0.0	30.4 ± 0.1	28.5 ± 0.0	39.9 ± 0.4	38.5 ± 0.0	30.2 ± 0.0
Ethanol volumetric productivity (g/L/h)^d^	0.53 ± 0.01	0.56 ± 0.00	0.56 ± 0.00	0.60 ± 0.01	0.59 ± 0.00	0.57 ± 0.00	0.62 ± 0.00	0.63 ± 0.00	0.59 ± 0.00	0.83 ± 0.01	0.80 ± 0.00	0.63 ± 0.00

^a^The experiments were carried out in YEP medium with 58.2 g/L glucose and 29.8 g/L xylose, 150 rpm, 30°C. Reactions proceeded for 48 h. Standard deviations from biological duplicate experiments are indicated.

^b^Yx/s, cell biomass yield on glucose was calculated based on maximum cell biomass during glucose fermentation and consumed glucose at that time.

^c^Specific xylose consumption rate was calculated based on time point 18 h.

^d^Ethanol volumetric productivity was based on the final concentration of ethanol.

The maintained cell viabilities of the Y2A and Y35 strains at elevated temperatures suggested the possibility that their thermotolerance may permit xylose fermentation at higher temperatures. While all the strains consumed all of the glucose from YEPXD (Figure 2A) , the Y1A, Y2A, and Y35 strains only consumed 5.1 g/L (17.0% of the initial xylose), 9.1 g/L (30.4%), and 13.1 g/L (43.5%) xylose, respectively by 48 h at 30°C (Figure 2B and Table 1). By comparison, the extensively engineered 424A strain consumed 29.5 g/L (99.0%) of the available xylose at 30°C. The Y1A strain, which lacks *SstiXYL123* genes for xylose metabolism, converted most of the consumed xylose into xylitol (Table 1). Interestingly, the Y35 strain maintained the same specific xylose consumption rate (total grams of xylose consumed per hour and per gram of dry cell weight) at 35 and 40°C (Table 1) whereas the specific xylose consumption rate for 424A at 40°C (0.046 g/h/g cell) was three times slower than that at 35°C (0.151 g/h/g cell). Furthermore, 424A displayed a greater than 3-fold reduction in percentage of total xylose consumption at 40°C (30.3%) compared to 35°C (96.5%). In contrast, Y35 had a 2-fold reduction at 40°C (24.5%) compare to 35°C (50.9%). This suggests the possibility that xylose metabolism by Y35 is more resistant to elevated temperatures relative to the 424A strain.

To assess whether the thermo-tolerance and xylose consumption properties of the GLBRC strains influenced ethanol production at higher temperatures, we determined the ethanol titers and metabolic yields from these fermentations (Figure 2C and Table 1). For ethanol titer, the 424A significantly outperformed both Y2A and Y35 at all temperatures, however, the 424A strain produced 21.6% lower ethanol titer at 40°C (30.2 g/L) compared to 35°C (38.5 g/L). The highest ethanol metabolic yields for Y2A and Y35 strains occurred at 40°C (84.2% and 83.2%, respectively), corresponding to the least xylitol and glycerol production at that temperature (Table 1). For 424A strain, the ethanol metabolic yield decreased slightly with increasing temperatures and more glycerol was produced at 35 and 40°C compared to 30°C. Together with our cell viability and xylose consumption rates at 35 and 40°C between Y35 and 424A strains, these ethanol production data suggest that the Y35 strain is relatively more tolerant to 40°C in YEPXD media than the 424A strain.

### Fermentation performance of *S.cerevisiae* Y35 in ACSH (SHF)

**Figure 3:**
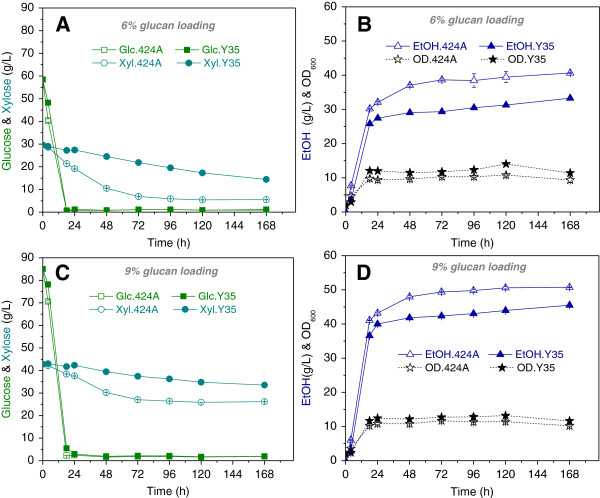
**Fermentation performance of Y35 and 424A in 6% and 9% glucan loading ACSH at 30°C. (A** and **B)**: 6% glucan loading; **(C** and **D)**: 9% glucan loading; **(A** and **C)**: Glucose and xylose consumption profiles; **(B** and **D)**: ethanol production and OD profiles.

As previously described here and elsewhere [7,8], degradation products from AFEX-pretreated biomass impair yeast fermentations. Thus, we examined the impact of two concentrations of inhibitors from AFEX pretreated corn stover on fermentation performance of the Y35 and 424A strains in 6% and 9% glucan loading ACSH at 30°C (Figure 3 & Table 2). The concentrations of major degradation products in 6% and 9% glucan loading ACSH are calculated based on the published data [13] and are shown in Additional file 2: Table S2. For both hydrolysates and strains, glucose was almost completely consumed in the first 18 h. After 168 h, Y35 consumed 51% and 21% of the available xylose and generated ethanol metabolic yields of 90.0% and 96.6% in the 6% and 9% glucan loading hydrolysates, respectively. In comparison, the 424A strain consumed 81% and 39% of xylose and generated ethanol metabolic yields of 97.6 and 99.7% in 6% and 9% glucan loading hydrolysates, respectively. The reductions in xylose consumption with 9% glucan loading ACSH were expected, given that higher glucan loadings result in higher hydrolysate inhibitor concentrations (Additional file 2: Table S2). These results indicate that the Y35 strain is similar to 424A in its relative tolerance to inhibitor concentrations in 6 and 9% glucan loading ACSH at 30°C.

**Table 2:** **SHF fermentation results summary of *S. cerevisiae *strains Y35 and 424A in 6% glucan loading and 9% glucan loading ACSH at 30°C**^**a**^

	**Y35 (6%)**	**Y35 (9%)**	**424A (6%)**	**424A (9%)**
Xylose consumption	51% ± 0.0	21% ± 0.0	81% ± 0.0	39% ± 0.0
Final xylose (g/L)	14.4 ± 0.17	33.5 ± 0.16	5.53 ± 0.11	26.2 ± 0.09
Yx/s^b^	0.10 ± 0.00	0.07 ± 0.00	0.08 ± 0.00	0.06 ± 0.00
Xylitol (g/L)	5.0 ± 0.1	3.1 ± 0.1	0.7 ± 0.0	0.7 ± 0.0
Glycerol (g/L)	3.8 ± 0.1	5.6 ± 0.0	5.0 ± 0.0	6.0 ± 0.1
Specific xylose consumption rate (g/h/g cell), 18 h^c^	0.020 ± 0.001	0.016 ± 0.001	0.076 ± 0.003	0.051 ± 0.000
Sugar consumption (%)^d^	82.3% ± 0.2%	72.3% ± 0.1%	92.7% ± 0.1%	78.1% ± 0.1%
Ethanol metabolic yield (%)	90.0% ± 1.7%	96.6% ± 0.3%	97.6% ± 0.3%	99.7% ± 0.4%
Final Ethanol concentration (g/L)	33.3 ± 0.5	45.5 ± 0.1	40.7 ± 0.2	50.7 ± 0.1
Ethanol volumetric productivity (g/L/h), 48 h^e^	0.61 ± 0.00	0.87 ± 0.00	0.77 ± 0.00	1.00 ± 0.01

^a^The experiments were carried out at 150 rpm and 30°C. Reactions proceeded for 168 h. Errors presented here were standard deviation of triplicate experiments.

^b^Yx/s, cell biomass yield on glucose was calculated based on maximum cell biomass during glucose fermentation and consumed glucose at that time.

^c^Specific xylose consumption rate was calculated based on time point 18 h.

^d^Sugar consumption was calculated based on available monomeric glucose and xylose for fermentation.

^e^Ethanol volumetric productivity was based on the 48 h results.

### SSCF performance of *S.cerevisiae* Y35 on ACS

The thermo- and AFEX inhibitor-tolerant properties of the Y35 strain suggested that it may fare well in SSCF of ACS with *Trichoderma* enzymes that optimally perform at temperatures above the standard 30°C for *S. cerevisiae*[5]. Therefore, we performed SSCF experiments at both 6% and 9% glucan loadings by first pre-hydrolyzing ACS for 6 h at optimal conditions for enzymatic liquefaction, followed by adjustment to pH 5.5 and inoculation of Y35 or 424A strain and then incubation at 30°C or 35°C. During pre-hydrolysis, approximately 35 g/L glucose and 21 g/L xylose were released at 6% glucan loading and approximately 45 g/L glucose and 29 g/L xylose were released at 9% glucan loading.

**Figure 4:**
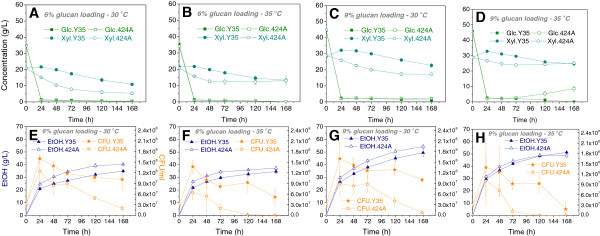
**SSCF performance comparison of Y35 and 424A on ACS at 6% and 9% glucan loading and 30 and 35°C. (A-D)**: glucose and xylose consumption profiles; **(E-H)**: ethanol production and cell viability profiles.

After inoculation of yeast cells, glucose was rapidly consumed to completion after 24 h for all cases (Figure 4A-D). However, the glucose concentration increased from 72 h to 168 h with 424A at 9% glucan loading and 35°C (Figure 4D). Typically, the glucose concentration during SSCF after 24 h remained low as the yeast rapidly consumed the free glucose (Figure 4A-C). This increase in glucose concentration by 424A likely occurred due to a slower glucose consumption rate than the enzymatic glucose release rate, which may have been due to the low cell viability under those conditions (Figure 4H). In contrast, the cell viability of Y35 remained high under the same conditions (Figure 4H) and accumulation of glucose was not seen (Figure 4D). In addition, 424A consumed significantly less xylose and produced significantly less ethanol at 35°C relative to 30°C at both 6% and 9% glucan loadings (*p* < 0.05, Figure 4). The cell viabilities of 424A dramatically decreased after 72 h under those conditions. In contrast, the SSCF performance of Y35 showed no significant differences between 30 and 35°C at 6 and 9% glucan loading ACS (Figure 4), respectively. Y35 also showed greater cell viabilities compared to 424A during SSCF (Figure 4E-H), particularly during later fermentation times. Interestingly, although Y35 produced significantly lower ethanol titers compared to 424A in SSCF at 30°C, Y35 produced nearly equal or higher ethanol titers than 424A at 35°C with 6% and 9% glucan loading ACS, respectively (Table 3). These results indicate that the Y35 strain compares favorably to the industrial benchmark 424A strain in SSCF with ACS at 35°C, and validates our approach to screen and select yeast strain backgrounds with phenotypic properties relevant to specific hydrolysate conditions and industrial processes.

**Table 3:** **Summary of SSCF results on ACS using Y35 and 424A at two biomass solids loadings (6% glucan loading and 9% glucan loading) and at 30 or 35°C**^**a**^

	**Y35 (6%-30°C)**	**Y35 (6%-35°C)**	**424A (6%-30°C)**	**424A (6%-35°C)**	**Y35 (9%-30°C)**	**Y35 (9%-35°C)**	**424A (9%-30°C)**	**424A (9%-35°C)**
Final xylose conc. (g/L)	10.9 ± 0.8	13.3 ± 2.5	5.4 ± 0.6	13.1 ± 1.4	22.70 ± 1.35	24.69 ± 1.27	17.2 ±0.8	25.2 ± 0.6
Xylitol (g/L)	4.7 ± 0.5	4.4 ± 1.0	0.4 ± 0.0	0.6 ± 0.2	2.9 ± 1.0	4.9 ± 1.4	0.0 ±0.0	0.4 ± 0.1
Glycerol (g/L)	5.4 ± 0.4	5.5 ± 0.2	5.9 ± 0.2	6.3 ± 0.5	7.4 ± 0.4	6.8 ± 0.6	7.5 ±0.4	7.7 ± 0.4
Final Ethanol conc. (g/L)	34.9 ± 0.8	34.6 ± 1.7	40.3 ± 0.2	37.3 ± 1.0	49.5 ± 2.9	51.3 ± 0.4	54.4 ±1.3	48.8 ± 1.7
Monomeric sugar conversion^b^	72.5% ± 0.8%	73.7% ± 1%	71.8% ± 0.4%	73.6% ± 0.7%	68.7% ± 2.4%	71.7% ± 0.4%	69.8% ±1.5%	71.7% ± 1.7%
Sugar consumption^c^	87.0% ± 1.1%	84.9% ± 3.1%	93.6% ± 0.9%	84.6% ± 1.7%	80.8% ± 1.9%	80.3% ± 0.8%	84.7% ±0.7%	74.0% ± 1.5%
Ethanol volumetric productivity (g/L/h), 48 h^d^	0.52 ± 0.02	0.56 ± 0.00	0.63 ±0.01	0.65 ±0.01	0.69 ±0.02	0.76 ± 0.02	0.78 ± 0.02	0.82 ± 0.02

^a^SSCF experiments were carried out in 250 ml baffled flasks with working weight of 100 g. Shaking speed, temperature and initial pH were 180 rpm, 30°C and 5.5, respectively.

^b^Monomeric sugar conversion was calculated based on the ethanol concentration and the remaining monomeric glucose and xylose in the fermentation broth after 168 h with the assumption of ethanol metabolic yield the same as SHF.

^c^Sugar consumption was calculated based on available monomeric glucose and xylose for fermentation.

^d^Ethanol volumetric productivity was based on the 48 h results.

## Discussion

Simultaneous saccharification and co-fermentation at high solids loadings is a desirable process in the biofuels industry due to the resulting higher ethanol titer, higher productivity and less water use [33]. However, high solids loading processes result in elevated levels of inhibitors, which have profound impacts on xylose fermentation. While shorter SSCF process times can be achieved with faster biomass hydrolysis rates by *Trichoderma* cellulases at relatively higher temperatures [5], any elevation in temperature above 30°C ultimately impacts yeast fermentation efficiency and productivity [8]. Here, we have described a phenotypic screening approach to identify and select yeast strains with innate tolerance to the inhibitory conditions present during the SSCF of high solids loading ACS at elevated temperatures. By characterizing the growth properties of 108 unique wild and domesticated *S. cerevisiae* strains in high solids loading ACSH at 40°C, we identified 11 strains (CBS7960, DBVPG6040, GLBRCY0, IL-01, NC-02, PE-2, PW5, T7, UWOPS83-787.3, UWOPS87-2421 and YJM451) that grew faster relative to other strains (Figure 1). The CBS7960, DBVPG6040, NC-02, PE-2, PW5, and T7 strains were isolated from industrial fermentation plants while the UWOPS83-787.3 and UWOPS87-2421 strains were both isolated from cacti but in distinct geographical locations (Additional file 1: Table S1). This suggests the possibility that the phenotypic properties of strains were partially selected for by their environmental conditions. The GLBRCY0 strain also displayed a relatively faster growth compared to the ATCC4124 strain, the ancestral parent of 424A, in 9% glucan loading ACSH at 40°C (Figure 1C-D). This suggests that engineered descendants of GLBRCY0 may have a relative advantage in simultaneously tolerating elevated temperature and degradation products compared to the 424A strain.

Since we had previously engineered the GLBRCY0 strain [27], we opted to further develop this genetic background to assess the validity of our approach. The engineered GLBRC strains (Y2A and Y35) could grow to higher cell densities in YEP medium at elevated temperatures (35 and 40°C) than 424A (Figure 2). In SHF experiments at 30°C (Figure 3 and Table 2), the 424A strain experienced a greater impact on specific xylose consumption rates between 6% and 9% glucan loading ACSH (0.076 and 0.051 g/h/g cell, respectively) compared to Y35 (0.020 and 0.016 g/h/g cell, respectively). These relative differences further suggested that the engineered Y35 strain retained some level of inhibitor tolerance on the fermentation of xylose from 9% glucan loading ACSH.

Taken together, the individual thermo- and inhibitor-tolerant properties of the Y35 strain suggested that Y35 could also tolerate the elevated temperature and inhibitor conditions present during SSCF of high solids loading ACS. Indeed, we found that while the 424A strain did not display significant differences between 30 and 35°C for xylose consumption and ethanol production on YEPXD medium (Figure 2B-C), its final ethanol titer and xylose consumption during SSCF of ACS at 35°C was significantly reduced compared to 30°C (Figure 4 and Table 3). In contrast, Y35 did not show significant differences in ethanol titer and xylose consumption between 30 and 35°C during SSCF. Moreover, Y35 achieved greater final ethanol concentration and percentage of total sugar consumed than 424A in SSCF at 35°C and 9% glucan loading (Table 3), supporting the notion that Y35 has greater tolerance to simultaneous elevated temperature and ACS degradation products. This tolerance is manifested as significantly greater cell viability for Y35 relative to 424A under these conditions (Figure 4, lower graphs), which likely allowed Y35 to continue the fermentation of sugars later in the process when 424A was no longer viable. At present, it is unclear what molecular mechanisms or specific genes are responsible for the relatively higher tolerance to AFEX inhibitors and elevated temperature by the Y35 strain compared to 424A. Future comparative gene expression or genome sequence studies may shed light on this.

Sugar inhibition is the major factor impeding sugar yield during enzymatic hydrolysis in SHF [33]. In SSCF, sugar inhibition is removed via fermentation and ethanol has lower inhibitory effect on enzymes compared to sugars [4]. Therefore, in most cases, SSCF yields higher ethanol titers than SHF [5,6], especially at higher solids loadings, even though SSCF performs at a temperature lower than the optimum of the enzymes. For instance, SSCF with Y35 at 9% glucan loading and 30°C yielded 49.5 g/L ethanol (Table 3) while SHF with separate enzymatic hydrolysis at 50°C and fermentation at 30°C using Y35 produced 45.5 g/L ethanol (Table 2). Conducting SSCF at a higher temperature improves enzymatic hydrolysis rates during the process [5] and hence enhances ethanol production rates. For example, during 9% glucan loading SSCF, Y35 produced a higher ethanol titer at 35°C compared to 30°C at a given time (Figure 4G &H). This further suggests that a stress-tolerant fermentation microbe could make the SSCF process more promising for industrial use.

## Conclusions

Here, we provide the phenotypic data comparing the growth profiles of 108 wild and domesticated *S. cerevisiae* strains grown in ACSH at elevated temperature. One wild strain, designated GLBRCY0, was one of the 11 strains that could grow in 9% glucan loading ACSH at 40°C. The Y35 strain, which was generated by engineering xylose metabolism into the GLBRCY0 strain, was found to be tolerant to simultaneous elevated temperature and AFEX degradation products during high solids loading SSCF. It is important to note that even without substantial metabolic engineering and adaptation, the Y35 strain performed similarly as the well-developed industrial benchmark 424A strain under certain conditions. Additional genetic modifications may be able to improve its ability to ferment xylose. These results support the hypothesis that our phenotypic screening approach may enable the generation of new cellulosic ethanologens selected and tailored for specific pretreatment and hydrolysis methods, types of biomass or custom fermentation processes.

## Methods

### AFEX pretreated Corn Stover

Corn stover, provided by the Great Lakes Bioenergy Research Center (GLBRC), was milled before pretreatment and was passed through a 4 mm sieve. The milled corn stover was AFEX pretreated in a 5 gallon high pressure reactor [34]. AFEX pretreatment conditions used for this study include: ammonia to biomass loading 1.0 g/g dry biomass, water loading 0.6 g/g dry biomass, temperature 100°C and residence time 30 minutes. AFEX pretreated corn stover was used as is for enzymatic hydrolysis/fermentations with no washing, detoxification or nutrient supplementation. The pretreated corn stover has glucan, xylan and acid insoluble lignin contents of 38.0%, 23.8% and 20.4%, respectively.

### Enzymatic Hydrolysis

Enzymatic hydrolysis using a commercial enzymes mixture was conducted at glucan loadings of 6% (w/w) and 9% (w/w) (corresponding to 15.8% and 23.7% solids loading, respectively) in a 2.0 L baffled flask with 450 g total mixture at pH 4.8, 50°C, and 250 rpm. The mixture of commercial enzymes was composed of Ctec 2 (Novozymes, North Carolina, USA) 20 mg protein/g glucan, Htec 2 (Novozymes, North Carolina, USA) 5 mg protein/g glucan and Multifect pectinase (Genencor Inc, Palo Alto, USA) 5 mg protein/ g glucan.

After 96 h hydrolysis, hydrolysate was centrifuged at 12,000 rpm for 30 min to remove unhydrolyzed solids followed by sterile filtration using a 0.2 μm Stericup from Millipore, Massachusetts. The filtered hydrolysate was stored at 4°C before being used for fermentation.

### Native and engineered *S. cerevisiae* strains

Native *Saccharomyces cerevisiae* strains used in this study were obtained from USDA ARS Culture Collection and Dr. Cletus Kurtzman (USDA ARS, Peoria, IL), National Collection of Yeast Cultures (Norwich, UK), and Dr. Justin Fay (Washington University, Saint Louis, MO) and are described in Additional file 1: Table S1. Genetic engineering of the Y1A and Y2A strains has been described elsewhere [27]. To generate the Y35 strain with 2 integrated copies of the *SstiXYL123* cassette, the Y2A strain was sporulated in 1% potassium acetate pH 7 for 10 days at room temperature with shaking. Tetrads dissected were grown on YEPD (5 g/L yeast extract, 10 g/L peptone, 20 g/L dextrose) agar plates and incubated for 2 days at 30°C. In most instances, colonies were then re-struck onto YEPD agar, or YEPD agar with an α mating type tester strains spread on the plate surface, or YEPD + 200 ug/ml Geneticin (Invitrogen). Two tetrads, Y27D (α mating type) and Y26B (*a* mating type) were isolated. Each strain contained the *SstiXYL123* cassette as confirmed by polymerase chain reaction (PCR) and antibiotic resistance. Y27D was mated with Y26B to generate the diploid strain, Y35, as verified by loss of a single mating type. The *S. cerevisiae* 424A(LNH-ST) strain [18] was obtained from Dr. Nancy W. Y. Ho, Purdue University.

### Growth phenotyping of *Saccharomyces cerevisiae* strains

To compare the relative growth properties amongst strains, 108 *S. cerevisiae* strains were individually arrayed in two separate 96-well plates, which included duplicate wells of commonly-used lab strains BY4741, CEN.PK113-5D and CEN.PK2-1D on both plates to serve as control strains for assessing plate-to-plate variability. Strains were stored as frozen in 96-well plates at −80°C in YEPD (10 g/L yeast extract, 20 g/L peptone, 20 g/L dextrose) + 15% (v/v) glycerol. 48 h before phenotyping, frozen strains were thawed and 10 μl of cells were inoculated into 500 μl of YEPD media, or single colonies of individual engineered yeast strains were inoculated into 5 ml YEPD media + 200 μg/ml Geneticin, in the interior wells of a 96-deep well block (NUNC) with a multichannel pipettor. After inoculation, the deep well block was sealed with breathable tape (Axygen), covered with a lid and incubated in 30°C platform shaker. After 48 h of growth, 10 μl of saturated cultures from the deep well block were used to inoculate the interior wells of a standard 96-well plate (NUNC) containing 190 μl of YEPD, or AFEX corn stover hydrolysate for native strains, or YEP media with 2% xylose (YEPX) and 200 μg/ml Geneticin for engineered strains, while outer wells (rows A and H, columns 1 and 12) contained 200 μl sterile water.

Inoculated 96-well plates were placed in Tecan F500 or M1000 multimode plate readers maintaining an interior chamber temperature of 30 or 40°C. Plates were shaken for 10 sec and absorbance at 595 nm measured from each well every 10 minutes for approximately 24 h with no shaking. Background subtracted absorbance readings for each strain were analyzed using an automated program called GCAT (available for download at http://www.glbrc.org/gcat-vm/) that uses nonlinear regression using the Richards equation [35] or a logistic function [36], to report individual specific growth rates (T. Sato and Y. Bukhman, manuscript submitted). To control for stochastic biological and technical variability between independent experiments, averaged specific growth rates for each strain were normalized relative to the growth rates in YEPD at 30**°**C, which serves as a reference condition across independent biological replicates. Normalized growth rates from three independent biological replicates in YEPD, 6% ACSH or 9% ACSH at 40**°**C were averaged, binned into groups (0–0.2 = “No growth,” 0.2-0.4 = “Slow” growth, 0.4-0.8 = “Moderate” growth, >0.8 = “Fast” growth) and were hierarchically clustered with Spotfire (TIBCO).

### *In vitro* enzymatic activity assays

*In vitro* xylose reductase and xylitol dehydrogenase activities were performed as previously described with minor modifications [37,38]. Y1A, Y2A and Y35 strains were grown in YEPX to optical density at 600 nm (OD_600_) of 0.8, and then cells from 45 ml of culture were harvested, washed with 10 ml 0.85% NaCl and re-suspended in an equal volume of breaking buffer (100 mM MOPS, pH 7.0, and 1 mM β-mercaptoethanol). After suspending in breaking buffer, cells were transferred to a glass tube containing glass beads and were vortexed to break the cells. The resulting material was centrifuged and the supernatant (clear lysate) was used in the activity assays. Specific XR and XDH activities were measured in ranges of 7–45 and 40–250 mU activity/mg total protein, respectively, from Y2A and Y35 cell extracts in the presence of co-factors (NADH or NADPH for XR; NAD^+^ or NADP^+^ for XDH).

### Fermentations in YEP medium or hydrolysates

Yeast inocula were prepared in YEP medium with 50 g/L glucose in a 250 ml Erlenmeyer flask with a working volume of 100 ml. A frozen glycerol stock was used for inoculation with initial optical density (OD_600_) of the seed culture approximately 0.1. The culture was grown at 30 °C and 150 rpm under micro-aerobic conditions for 20–24 h.

Fermentations in ½ YEP medium (yeast extract 5 g/L and peptone 10 g/L) with 58.2 g/L glucose and 29.8 g/L xylose (YEPXD), as well as 6% glucan loading and 9% glucan loading hydrolysates were carried out in 125 ml Erlenmeyer flasks with a working volume of 50 ml, pH 5.5, temperature 30°C and shaking speed 150 rpm. The flasks were capped with rubber stoppers pierced with a needle. Fermentations were initiated with an OD_600_ of 2.0 through inoculation of yeast cell pellets obtained by centrifuge of seed culture [6].

Ethanol metabolic yield was calculated based on the theoretical ethanol yield from consumed glucose and xylose, which is 0.51 g ethanol/g glucose or xylose.

### Simultaneous saccharification and co-fermentation of ACS

SSCF were performed using identical glucan and enzyme loadings as in the enzymatic hydrolysis described above. Experiments were carried out in a 250 ml baffled flask with 100 g total mixture in a shaking incubator (Innova, New Brunswick, NJ). The biomass was first pre-hydrolyzed using all of the enzymes at pH 4.8, 50°C and 250 rpm for 6 h. After 6 h, the pH, temperature, and shaking speed were adjusted to 5.5, 30°C, and 180 rpm, respectively and yeast cells were inoculated at an OD of 2.0 to initiate the SSCF [6]. During all fermentations, the pH was passively maintained at around 5.2-5.5.

Glucose, xylose and ethanol concentrations in fermentation broth were analyzed using HPLC with a Biorad Aminex HPX-87H column as described previously [39]. Column temperature was maintained at 50°C. Mobile phase (5 mM H_2_SO_4_) flow rate was 0.6 mL/min.

### Measurement of viable cell density

Viable cell density was measured in colony forming unit (CFU) per ml. Fermentation samples were diluted using sterile water and 20 μL of each diluted sample was used for plating on YEP agar medium (25 g/L glucose and 25 g/L xylose). Dilution factor was varied to make sure the number of colonies on a single plate was between 20 to 100. During dilution, the sample solutions were vigorously vortexed to prevent cell clumping or adhering to the solid biomass. After 24 h incubation of the plates at 30°C, single colonies were counted and viable cell density was calculated accordingly. The standard deviations were generated from biological duplicates.

### Statistical analyses

For the determination of statistical significance, *t*-test was performed using Minitab15 Statistical Software (2006 Minitab Inc, Pennsylvania, USA). The statistically significant difference criterion was set as *p* < 0.05.

## Abbreviations

424A: *Saccharomyces cerevisiae* 424A(LNH-ST) strain; ACS: AFEX pretreated corn stover; ACSH: AFEX pretreated corn stover hydrolysate; AFEX: Ammonia fiber expansion; CFU: Colony forming unit; GLBRC: Great lakes bioenergy research center; HMF: 5-hydroxymethyl-2-furaldehyde; NAD: Nicotinamide adenine dinucleotide; NADH: Nicotinamide adenine dinucleotide (reduced form); NADP: Nicotinamide adenine dinucleotide phosphate; NADPH: Nicotinamide adenine dinucleotide phosphate (reduced form); OD600: Optical density at 600 nm; PCR: Polymerase chain reaction; S. cerevisiae: *Saccharomyces cerevisiae*; SHF: Separate hydrolysis and fermentation; SSCF: Simultaneous saccharification and co-fermentation; Ssti: *Scheffersomyces stipitis*; S. stipitis: *Scheffersomyces stipitis*; SstiXYL123: *S. stipitis XYL1, XYL2* and *XYL3* genes; XDH: Xylitol dehydrogenase; XK: Xylulokinase; XR: Xylose reductase; Y1A: *Saccharomyces cerevisiae* GLBRCY1A strain; Y2A: *Saccharomyces cerevisiae* GLBRCY2A strain; Y35: *Saccharomyces cerevisiae* GLBRCY35 strain; YEP: Yeast extract and peptone medium; YEPD: Yeast extract, peptone and dextrose medium; YEPX: Yeast extract, peptone and xylose medium; YEPXD: Yeast extract, peptone, xylose and dextrose medium.

## Competing interests

The authors declare that they have no competing interests.

## Authors’ contributions

MJ designed and supervised the execution of fermentation experiments, analyzed the data, coordinated this study and wrote the manuscript. CS and CG carried out the fermentation experiments and helped analyze the data. BB performed strain engineering and characterization. SS and RN performed enzyme activity assays. LW performed enzyme activity assays, analyzed data and participated in the manuscript writing. BD participated in the coordination and supervision of this study and edited the manuscript. VB coordinated this study, participated in the experimental design and data evaluation, and edited the manuscript. TS coordinated this study, designed and executed the strain phenotyping and engineering, and wrote the manuscript. All authors read and approved the final manuscript.

## Supplementary Material

Additional file 1: Table S1 Description of native *Saccharomyces cerevisiae* strains used in this study. Click here for file

Additional file 2 **Figure S1.** Growth and biochemical characterization of engineered GLBRCY0 strains on YEP media containing 2% xylose. Relative cell densities of Y1A (closed circles), Y2A (open squares) and Y35 (closed triangles) strains grown in YEPX media at 30°C (**A**) or 40°C (**B**). Background subtracted cell densities measured by 96-well plate readers were normalized relative to the cell density at 1 h after inoculation. Standard deviations were calculated from biological triplicates. Only time points every 30 minutes are shown for clarity. Relative *in vitro* specific xylose reductase (**C**) and xylitol dehydrogenase (**D**) activities were determined from the indicated strain extracts in the presence of co-factors (NADH or NADPH for XR; NAD^+^ or NADP^+^ for XDH). The bar graph indicates the relative specific xylose reductase and xylitol dehydrogenase activities expressed as a percentage of the specific activity from the Y2A strain with NADPH or NAD^+^, respectively. Average relative activities and standard deviations were determined from two independent biological replicates. **Table S2.** Concentrations of major degradation products in 6% and 9% glucan loading ACSH. The concentrations were calculated based on the data from ref. [13]. Click here for file
