# Supplementary material for: Phenotypic selection of a wild Saccharomyces cerevisiae strain for simultaneous saccharification and co-fermentation of AFEX™ pretreated corn stover
Source: Biotechnol Biofuels. 2013 Jul 27;6:108. doi: 10.1186/1754-6834-6-108 (PMC3729497; doi:10.1186/1754-6834-6-108)
Supplement: Additional file 2 — Figure S1. Growth and biochemical characterization of engineered GLBRCY0 strains on YEP media containing 2% xylose. Relative cell densities of Y1A (closed circles), Y2A (open squares) and Y35 (closed triangles) strains grown in YEPX media at 30°C (A) or 40°C (B). Background subtracted cell densities measured by 96-well plate readers were normalized relative to the cell density at 1 h after inoculation. Standard deviations were calculated from biological triplicates. Only time points every 30 minutes are shown for clarity. Relative in vitro specific xylose reductase (C) and xylitol dehydrogenase (D) activities were determined from the indicated strain extracts in the presence of co-factors (NADH or NADPH for XR; NAD+ or NADP+ for XDH). The bar graph indicates the relative specific xylose reductase and xylitol dehydrogenase activities expressed as a percentage of the specific activity from the Y2A strain with NADPH or NAD+, respectively. Average relative activities and standard deviations were determined from two independent biological replicates. Table S2. Concentrations of major degradation products in 6% and 9% glucan loading ACSH. The concentrations were calculated based on the data from ref. [13]. [file 1754-6834-6-108-S2.docx]

**Additional file 2**

**Phenotypic selection of a wild *Saccharomyces cerevisiae* strain for Simultaneous Saccharification and Co-Fermentation of AFEX^TM^ pretreated corn stover**

Mingjie Jin^1,2*^, Cory Sarks^1,2^, Christa Gunawan^1,2^, Benjamin D. Bice^3^, Shane P. Simonett^4^, Ragothaman Avanasi Narasimhan^3^, Laura B. Willis^3,4,5^, Bruce E. Dale^1,2^, Venkatesh Balan^1,2*^, and Trey K. Sato^3*^

^*^Co-corresponding authors

Mingjie Jin^1,2*^ E‐mail: [jinmingj@egr.msu.edu](mailto:jinmingj@egr.msu.edu)

Venkatesh Balan^1,2*^ E-mail: [balan@egr.msu.edu](mailto:balan@egr.msu.edu)

Trey K. Sato^3*^ E-mail: [tksato@glbrc.wisc.edu](mailto:tksato@glbrc.wisc.edu)

^1^Biomass Conversion Research Laboratory (BCRL), Department of Chemical Engineering and Materials Science, Michigan State University, 3900 Collins Road, Lansing, MI 48910.

^2^DOE Great Lakes Bioenergy Research Center, Michigan State University, East Lansing, MI 48824.

^3^DOE Great Lakes Bioenergy Research Center, University of Wisconsin-Madison, 1552 University Ave, Madison, WI 53726.

^4^Department of Bacteriology, University of Wisconsin-Madison, 1550 Linden Dr, Madison, WI 53706.

^5^U.S. Department of Agriculture, Forest Products Laboratory, 1 Gifford Pinchot Dr, Madison, WI 53726.

**Figure S1** Growth and biochemical characterization of engineered GLBRCY0 strains on YEP media containing 2% xylose. Relative cell densities of Y1A (closed circles), Y2A (open squares) and Y35 (closed triangles) strains grown in YEPX media at 30 °C (A) or 40 °C (B). Background subtracted cell densities measured by 96-well plate readers were normalized relative to the cell density at 1h after inoculation. Standard deviations were calculated from biological triplicates. Only time points every 30 minutes are shown for clarity. Relative *in vitro* specific xylose reductase (C) and xylitol dehydrogenase (D) activities were determined from the indicated strain extracts in the presence of co-factors (NADH or NADPH for XR; NAD^+^ or NADP^+^ for XDH). The bar graph indicates the relative specific xylose reductase and xylitol dehydrogenase activities expressed as a percentage of the specific activity from the Y2A strain with NADPH or NAD^+^, respectively. Average relative activities and standard deviations were determined from two independent biological replicates.

**
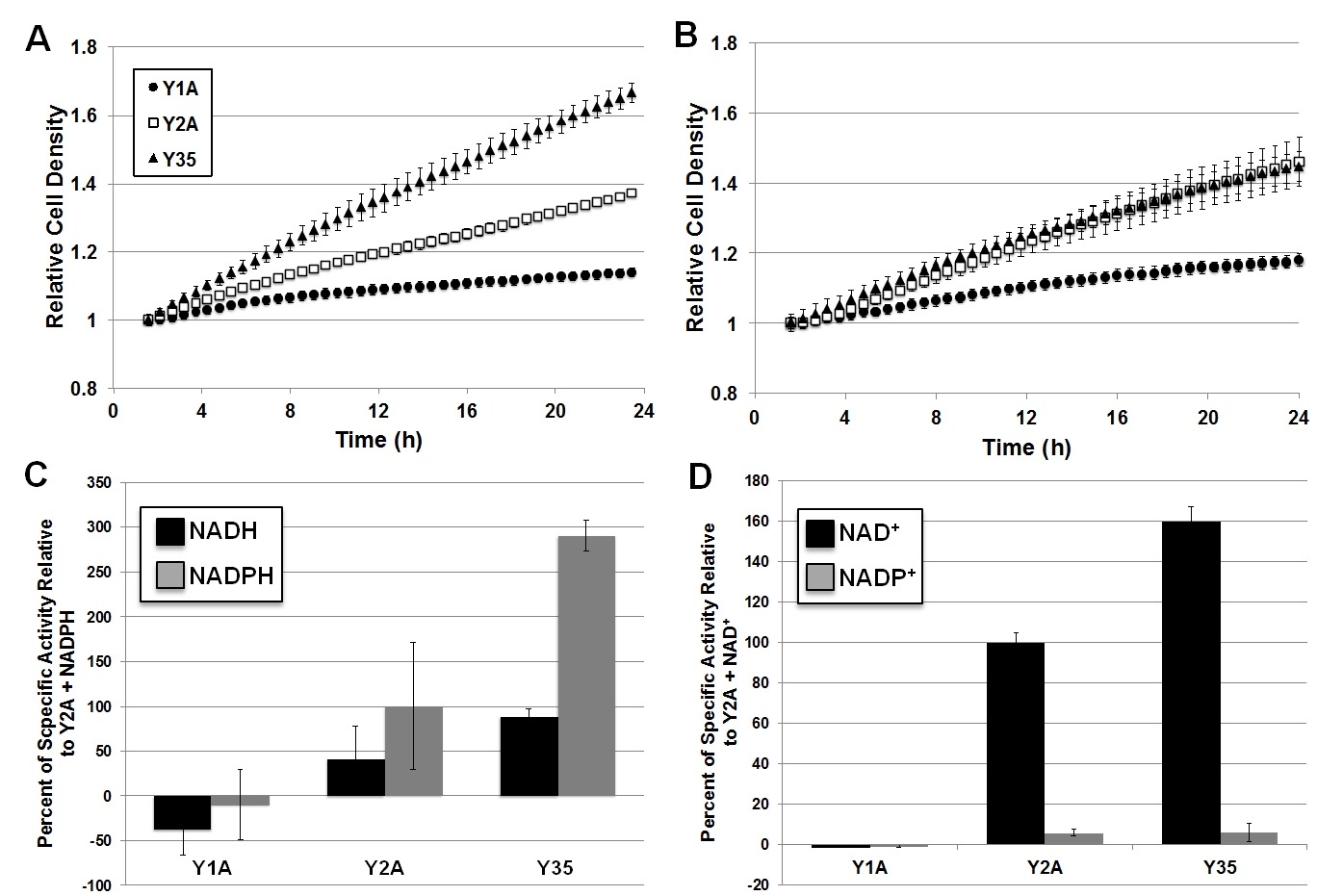
**

**Supplemental Table S2** Concentrations of major degradation products in 6% and 9% glucan loading ACSH. The concentrations were calculated based on the data from ref. (13)

| Degradation products | 6% glucan loading ACSH (g/L) | 9% glucan loading ACSH  (g/L) |
| --- | --- | --- |
| 5-Hydroxymethyl furfural (HMF) | 0.1126 | 0.1748 |
| Furfural | 0.0005 | 0.0008 |
| Lactic acid | 0.0558 | 0.0866 |
| Aconitic acid | 0.6654 | 1.0326 |
| Acetic acid | 0.8087 | 1.2550 |
| Formic acid | 0.1600 | 0.2483 |
| Levulinic acid | 0.0042 | 0.0065 |
| Acetamide | 4.3929 | 6.8166 |
| Total phenolic amides | 2.5896 | 4.0184 |
| Toal pyrazine and imidazole derivatives | 0.1658 | 0.2573 |
| 4-hydroxybenzaldehyde | 0.0163 | 0.0253 |
| Vanillic acid | 0.0081 | 0.0125 |
| Vanillin | 0.0342 | 0.0531 |
| Syringic acid | 0.0088 | 0.0136 |
| p-coumaric acid | 0.1895 | 0.2940 |
| Ferulic acid | 0.0181 | 0.0280 |
| Benzoic acid | 0.0016 | 0.0025 |
| 4-Hydroxyacetophenone | 0.0007 | 0.0011 |
| Syringaldehyde | 0.0019 | 0.0030 |
| Caffeic acid | 0.0009 | 0.0014 |
| Vanillic acid | 0.0081 | 0.0125 |
| 3-Hydroxy-4-methoxycinnamic acid | 0.0074 | 0.0114 |

(Chundawat et al, Bioresource Technology 2010, 101:8429-8438.)
